# Supplementary material for: Attention and social communication skills of very preterm infants after training attention control: Bayesian analyses of a feasibility study
Source: PLoS One. 2022 Sep 22;17(9):e0273767. doi: 10.1371/journal.pone.0273767 (PMC9499320; doi:10.1371/journal.pone.0273767)

SUPPLEMENTARY MATERIAL

ATTENTION AND SOCIAL COMMUNICATION SKILLS OF VERY PRETERM INFANTS AFTER TRAINING ATTENTION CONTROL: BAYESIAN ANALYSES OF A FEASIBILITY STUDY

Index:

Section 1: Information about Ethical approval and participants' informed consent;
Section 2: Completion and Engagement in the Intervention;
Section 3: Description of the Screen-Based Attention Tasks;
Section 4: Description of the Naturalistic Tasks;
Section 5: Introduction to Bayesian Analyses;
Section 6: Summary Scores Used in Analyses;
Section 7: Bayesian Regression Models;
Section 8: Distributions of d scores by outcome.

Section 1

Information about Ethical approval and participants' informed consent

This study was reviewed and approved by the Health and Social Care Research Ethics Committee A (HSC REC A), Office for Research Ethics Committees Northern Ireland (ORECNI), on 09 March 2018, REC Reference: 18/NI/0010; IRAS Project ID: 237537. The study has therefore been performed in accordance with the ethical standards laid down in the 1964 Declaration of Helsinki and its later amendments, as well as national laws concerning data protection.

Caregivers of infants enrolled in the study provided written informed consent to take part in the study before initiating the study. A blank consent form is provided in the following page.

The sponsor of the study was Governance, Ethics and Integrity, Queen's University Belfast, University Road, Belfast, BT7 1NN, UK.

The trial was retrospectively registered at Clinical Trials Protocol Registration and Results System (clinicaltrials.gov). Registration ID: NCT03896490.

The study categorised infants for their prematurity at birth, based on their gestational age. Eligible participants were infants born very preterm (between 28 and less than 32 weeks of gestation) residing in Northern Ireland. Participants were contacted before they were 12 months old from conception, with the aim that they would have started the study when aged approximately 1 year at the start of the study, age corrected for prematurity. Exclusion criteria were: significant visual and/or hearing disabilities; congenital anomalies that may impact on infants' cognitive and sensory-motor development; a diagnosis of Cerebral Palsy; current or recent participation in a trial which may interfere with this study (e.g. by affecting concentration abilities) or represented a significant burden for the family.

Participants were identified by collaborating neonatology practitioners in hospitals within the Belfast, South Eastern, and Northern Trust in Northern Ireland. Practitioners acted as gatekeepers and ensured that information on the study was passed to families of infants that were eligible to take part and did not meet any exclusion criterion. Interested parents contacted the research team to receive more details about the study and decide whether to take part or not. If parents agreed to take part, the research team documented their consent in writing and agreed an appointment. Furthermore, a local charity for families of premature children passed the information on the study to eligible parents, who could decide to contact the research team if they were interested. Parents who received information about the study from the charity were asked to consult with one of the collaborating neonatologists to ensure their child did not meet any exclusion criterion.

In the analyses we did not control for confounding variables such as socioeconomic status, nutrition, environmental exposures. The only variable controlled for was age at the time of infants' completion of the post-test assessments (see Methods).

Figure S1: Blank Participant Consent Form

Consent Form ACT v5 05.01.2018 IRAS Project ID: **237537**

University Logo

CONSENT FORM

A feasibility study of the Attention Control Training (ACT) intervention amongst very preterm (VP) infants.

Please **initial**

1. I confirm that I have read and understand the information sheet dated 15.02.2018 (version 7) for the above study and have had the opportunity to ask questions.	
2. I understand that the participation of my child and I is voluntary and that I am free to withdraw at any time, without giving any reason. I understand that if I decide to leave the study, I can ask all the data collected about me and my baby to be destroyed.	
3. I agree to my child and myself being video-recorded during the study tasks. I understand these video-recordings will be only accessible to members of the research team, and will be only used for study purposes.	
4. I understand that information collected during the study will remain anonymous. I understand the only exception to this is if there are circumstances that can put me or my baby at risk.	
5. I understand that data presented during the study will be anonymised and will not include information that may identify me or my baby. I agree that data may be looked at by members of the research team for study purposes.	
6. I agree to take part in this study.	

____________________________ ____________ _____________________

Name of participant Date Signature

____________________________ ____________ _____________________

Name of Person taking consent Date Signature

For office use only: Study ID ____ ____ _____

Section 2

Completion and Engagement in the Intervention

In this section we provide more information concerning the number, duration, and characteristics of the interventions and control procedures completed by the infants in the study. We provide this information to complement the information provided in the present manuscript, but we refer to another paper we have published for more details (see Perra et al., 2021).

Overall, the 10 participants included in the study completed 33 visits, of which 17 were interventions completed by the *n* = 5 infants in the intervention group, and 16 were control procedures completed by the *n* =5 infants in the control group. Our procedure allowed parents to opt to receive the intervention or control procedure in their own house (in order to facilitate participation). Only one infant in the intervention group received the intervention at home in 2 visits out of 3 completed ones. Two infants in the control group completed all the visits at home, for a cumulative total of 6 visits. The same material and equipment and the same procedure was used in the lab and home delivery of the intervention/control procedure: The results of the feasibility study published (Perra et al., 2021) suggested infants were in an alert and calm state most of the time during the delivery of these procedures at home.

Training tasks have been described in a previous paper (Perra et al., 2020): these involved training goal maintenance (e.g. maintaining attention on a moving target); ability to identify a target among distracters; short term memory of objects embedded in scenes. Based on previous studies, we deemed a training task to have been completed if it lasted for at least 240 s. Overall, infants in the training group completed 4.65 tasks per session (SD = 1.62) for an average duration of completed tasks of 24.59 min per session (SD = 9.39). Infants in the control group completed 5.19 tasks pr session on average (SD = 1.54), for an average duration of sessions equal to 23.46 min (SD = 8.88). In our previous publication, we emphasised that the average duration of the training sessions completed by VP infants in this study was remarkably similar to that of term infants in previous studies (see Perra et al., 2021).

Further evidence of infants' engagement with the training was provided by performance during the training tasks. We reasoned that if infants were engaging in the training, they would display improvements in key performance indicators during the training tasks (e.g. shorter reaction times when infants had to fixate a pre-defined target among other targets). Results of multilevel analyses considering training sessions nested within infants

indicated significant improvements of performance across different types of tasks administered (see Perra et al., 2021, for details).

Perra, O., Wass, S., McNulty, A., Sweet, D., Papageorgiou, K., Johnston, M., Patterson, A., Bilello, D., & Alderdice, F. (2020). Training attention control of very preterm infants: protocol for a feasibility study of the Attention Control Training (ACT). *Pilot and Feasibility Studies*, *6*(1), [17]. https://doi.org/10.1186/s40814-020-0556-9

Perra, O., Wass, S., McNulty, A., Sweet, D., Papageorgiou, K. A., Johnston, M., Bilello, D., & Alderdice, F. (2021). Very preterm infants engage in an intervention to train their control of attention: results from the feasibility study of the Attention Control Training (ACT) randomised trial. *Pilot and Feasibility Studies*, *7*, [66]. https://doi.org/10.1186/s40814-021-00809-z

Perra, O., Wass, S., McNulty, A., Sweet, D., Papageorgiou, K. A., Johnston, M., Bilello, D., Patterson, A., & Alderdice, F. (2021). Correction to: Very preterm infants engage in an intervention to train their control of attention: results from the feasibility study of the attention control training (ACT) randomised trial. *Pilot and Feasibility Studies volume 7, Article number: 201*. https://doi.org/ 10.1186/s40814-021-00943-8

Section 3

Description of the Screen-Based Attention Tasks

The following tasks were administered to all infants before the delivery of the training/control procedure in Visit #1, and approximately one week after the end of the training/control procedure in Visit #5. These tasks were presented in a random computer-generated order, with the only caveat that, out of necessity, the Visual Paired Comparison task always followed the Sustained Attention one. The tasks were presented in two sessions, interspersed by the naturalistic tasks described in another section.

The screen-based tasks assessed different aspects of attention and cognitive control compared to those targeted by the training, thus testing *proximal* transfer of effects. In order to maintain infants' engagement, different short video-clips were presented between tasks to attract infants' attention on the screen. The tasks are described in more details in what follows, providing examples of stimuli used.

**Sustained attention**<br><br>A still image of a child's face on a white background was presented in silence. The blinded Assessor watching a live video feed of the child through a webcam mounted on top of the screen, coded whether the child was looking at the screen using a key press. When the child looked away for 1 s or more, this marked the end of the trial. The same image was then presented again until the child completed two consecutive looks at less than 50% of the longest look displayed: this was the habituation criterion (Colombo & Mitchell, 2009). If the child had not met the habituation criterion within 120 s of accumulated looking time or within 12 trials, the trial was aborted, and the block excluded.<br>**Outcomes of interest**: Outcome measures were *peak look*, calculated as the duration in seconds of the single longest unbroken look toward each stimulus, and *looks to criterion*, calculated as the number of looks required to fulfil the habituation criterion (Colombo & Mitchell, 2009). In line with other studies (e.g. Wass, Cook, & Clackson, 2017) in order to account for asymmetrical distributions, in analyses we used the *reciprocal* of the number of looks to criterion: thus, a higher score indicated *fewer* looks necessary to reach criterion and, therefore, *better* performance.	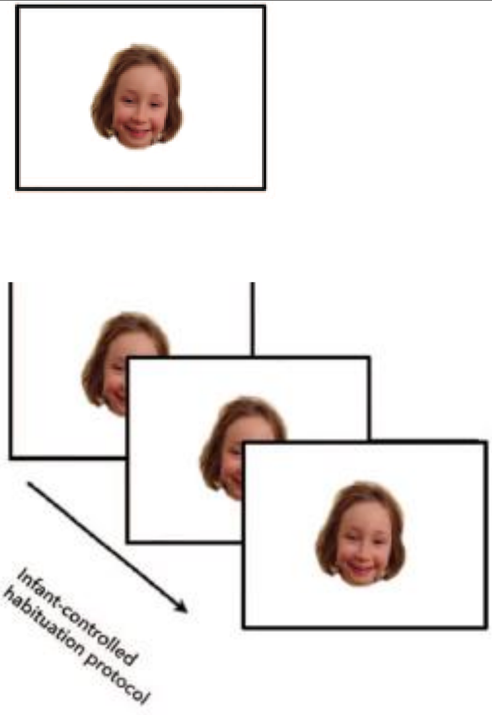

**Visual Paired-Comparison (Visual recognition Memory)**<br>The same familiar image from the previous task was presented concurrently with a novel image over three trials. The images were presented in one Left-Right order for 8,000 ms, successively swapping the order for the subsequent 8,000 ms.<br>**Outcomes of interest**: Proportion looking to the novel target was calculated as the time spent viewing the unfamiliar target, divided by the time spent viewing both the familiar and the unfamiliar target combined.	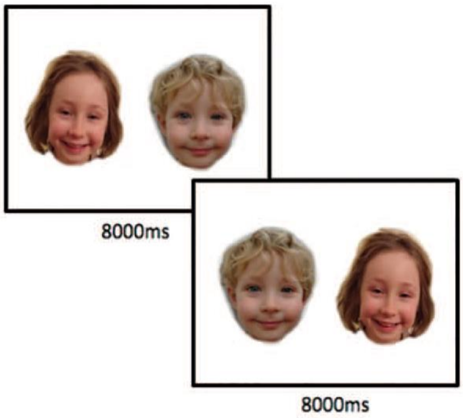
**Gap–overlap (Disengagement of Attention).**<br>This task was presented in three blocks. The first two blocks lasted 20 trials, while the third continued until 12 usable trials per condition had been collected, or 80 trials had been presented, or the infant became inattentive.<br>After fixating a central target (CT), following a variable Inter-Stimulus Interval (ISI) a lateral target (LT, a cartoon cloud) appeared to either the left or right; when the infant fixated the LT, a brief audio-visual display was presented on the screen. Three conditions were used:<br>*Gap* – CT disappeared 200 ms *before* LT appears;<br>*Baseline* – CT disappeared *concurrently* with LT appearance;<br>*Overlap* – CT remained on screen *with* LT appearance.<br>The order of trials was randomised between conditions.<br>**Outcomes of interest**: The key variable of interest was the reaction time (RT), namely the time elapsed between LT appearance and the reported position of gaze leaving the central fixation area. Reaction times less than 100 and greater than 2000 ms were excluded. Average reaction times were calculated by first averaging the reaction times obtained across the three separate conditions, and then combining the log transformed averages to create a final average. Following work by Elsabbagh and colleague's procedure (Elsabbagh et al., 2009), disengagement latencies were calculated as the participant's average reaction time in the overlap condition subtracted from their average reaction time in the baseline condition.	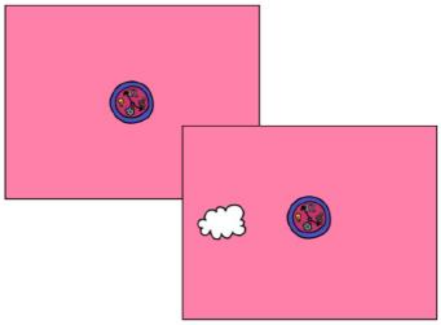
**Information Density Preference**<br>Six blocks were presented at different times during the testing session (3 conditions x 2 blocks for each condition). Each block lasted 100 seconds, and featured 4 images that were presented in sequence. The images	

ranged from a picture of a building to a colourful drawing of children and were the same for all infants. In condition 1, 'slow', each picture was presented once, for 25 s, during the block. In condition 2, 'medium', each picture was presented for 10 s. After each picture had been presented once, the sequence of 4 pictures was repeated. In condition 3, 'fast', each picture was presented for 1 s. After each picture had been presented once, the sequence of four pictures was repeated.<br>Look duration was measured by the Assessor manually coding the infants looks to and away from the screen using a key press.<br>**Outcomes of interest**: The main outcome was *Attention Capture,* defined as the difference in looking time in seconds to the 'fast', more salient and attention-eliciting presentation, minus the looking time to the 'slow', less salient presentation: increased looking times to the 'slow' presentation was considered to indicate infants' increased control over attention, whereby they were able to maintain attention to less attention-eliciting and salient presentations.	

References

Colombo, J., & Mitchell, D. W. (2009). Infant visual habituation. Neurobiology of Learning and Memory, 92(2), 225–234. https://doi.org/10.1016/j.nlm.2008.06.002

Elsabbagh, M., Volein, A., Csibra, G., Holmboe, K., Garwood, H., Tucker, L., and Johnson, M. H. (2009). Neural Correlates of Eye Gaze Processing in the Infant Broader Autism Phenotype. Biological Psychiatry, 65(1), 31–38. https://doi.org/10.1016/j.biopsych.2008.09.034

Wass, S.V., Cook, C., Clackson, K. (2017) Changes in behavior and salivary cortisol after targeted cognitive training in typical 12-month-old infants. *Developmental Psychology 53(5)*, 815-825. doi: 10.1037/dev0000266.

Section 4
Description of the Naturalistic Tasks

In the pre- and post-test assessments we also administered a series of naturalistic tasks delivered using structured observations. The purpose of these tasks was to observe infants' focused attention, social communication skills, and other aspect of infants' behaviour control and regulation in contexts more akin to every-day situations. Our aim was to investigate *distal* transfer of effects, namely, whether trained infants displayed improvements in socio-cognitive skills beyond attention lab tasks. We also collected a parental assessment using a validated questionnaire.

Four pseudo-random sequences of presentation that included interspersed screen-based and naturalistic tasks had been generated and were deployed counterbalanced between and within infants. The only conditions that we maintained constant were: (a) that the "Free Play" task was always first in the testing sessions, in order to allow for parents and infants to settle in and familiarise with the testing environment; (b) that the Lab-TAB Toy in the Box task was the last task administered, so that infants' protest in this task did not interfere with the other tasks.

The naturalistic tasks were video recorded for further analyses using two CCTV cameras on opposite sides of the room. In most of the task the infant sat on the parent's lap in front of a desk and opposite the blind Assessor. One camera captured the blinded Assessor and the other camera captured the infant and the parent. The two outputs from the cameras were recorded synchronously and saved on a laptop using Mangold® VideoSincPro software. Coding of recorded tasks was carried out by a coder blinded to infants' group allocation using Mangold® Interact coding software (version 18).

Lab-Tab Tasks: Orientation

The Orientation task from the Lab-Tab (Gagne, Van Hulle, Aksan, Essex, & Goldsmith, 2011; Planalp, Hulle, Gagne, & Hill Goldsmith, 2017) was administered to all infants. The Assessor introduced the task to the infant's parent saying: "In this task we are interested to see how children manipulate a set of blocks. Please allow [name of child] to play with the blocks on his/her own. If [name of child] seeks your involvement you may say 'yes, you play with it'. If the blocks have fallen on the floor or out of reach, and you can reach them, please put them back on the table in front of your child". The Assessor then presented a set of wooden blocks and said "Here are some blocks for you to play with", then addressed the parent saying: "I'll be 3 minutes", and left the room, watching the session through the

cameras recording in the room. The task provided an assessment of infants' orienting and focused attention skills in a controlled naturalistic setting.

Coding and Reliability

A coder blind to group allocation coded the infants' direction of gaze. Gaze direction categories indicated whether the infant was looking at the wooden blocks, at the parent, or somewhere else. The three-minute trial was then divided into intervals of 10 s. The coder watched each interval and produced a rating for infants' *intensity of facial interest*, and *parental interference*. Infants' intensity of facial interest was rated from 0 (no interest) to 2 (definite indication of facial interest) according to the peak intensity of infants' facial interest demonstrated during the 10 s interval. Parental interference was rated in a scale from 0 (no interference) to 2 (parent actively encouraging child to attend or manipulate blocks).

A second coder scored all the sessions recorded to check inter-reliability. The agreement was satisfactory in all the categories: 82% and $\kappa = 0.74$ for *direction of gaze*; 89% and $\kappa = 0.79$ for *intensity of facial interest*; and 87% and $\kappa = 0.75$ for *parental interference*.

Outcome in Analyses

The outcome used in analyses was the average of infants' intensity of facial interest across the 10 s periods of the task. Ratings of parental interference were low and similar across the two groups (0.38 and 0.48 at pre-, and 0.51 and 0.52 at post-test for the controls and trained infants respectively), therefore we did not control for this variable.

Object free play

The parent was also asked to play with the infant for 4 minutes, while we recorded these sessions. The Assessor told the parent: "We want to see how (baby's name) behaves in typical game situations. Therefore, can you please play with (baby's name) as you would do at home if you had some time together? There are some toys on the table that you can use if you want. We will video-record you and (baby's name) for four minutes, but we won't interfere. If (baby's name) becomes

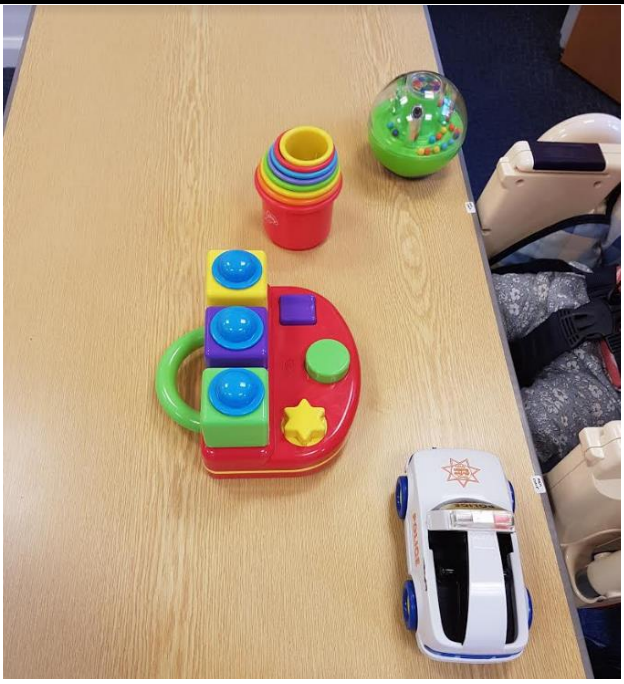

tired or needs a break, just ask us to stop." The infant sat on an infant chair in front of the table opposite the parent. Four toys (a toy car, a pop-up toy, a coloured ball, and a set of stacked cups) were presented in front of the infant on the table in the way pictured in the picture provided.

The recording from these sessions was used to investigate infants' ability to focus attention on objects and on the parent, as well as abilities to coordinate attention between objects and the parent. The main outcome of interest was the percentage of time infants spent looking at the objects.

Coding and Reliability

A researcher blind to infants' group allocation coded all the recorded tasks continuously using exhaustive and mutually-exclusive categories that indicated the focus of attention of infants during the task (objects, parent, or other). Additional codes were used to indicate if infant's gaze was occluded/not visible, or the eyes closed. If the infant looked at one of the objects, the coder also coded which of the four objects in front of the infant she was attending to. In order to check coding reliability, a second researcher coded a random sample of the recorded tasks (n=7), representing over a third of all the recorded tasks. The agreement was excellent, being 95% for the direction of gaze and 90% for the specific object being attended, $\kappa = 0.88$ and $\kappa = 0.86$ respectively.

Outcome in Analyses

The outcome used in analyses was the cumulative proportion of time infants looked at any of the objects on the total duration of the session (excluding times when infants' gaze was not visible or occluded, e.g. by the parent in front of her). This outcome was considered to index infants' focused attention.

Infant Behavior Questionnaire (IBQ)

We asked parents to complete the very short form of the Infant Behavior Questionnaire (Putnam, Helbig, Gartstein, Rothbart, & Leerkes, 2014) before the first session and then at the conclusion of the study. The questionnaire asks a series of questions concerning the infants' behaviour as observed by parents in daily situations. Questionnaires were double entered by a researcher, with discrepancies checked and reconciled. The questionnaire provides a validated assessment of infants' temperamental traits. In particular, we were interested in traits that are related to attention and behaviour control.

Outcome in Analyses

The main outcome was the "Effortful Control" dimension, which indicates infants' enjoyment of low-intensity activities (e.g. being held or rocking), focused attention, and inhibitory control.

Tasks from the Early Social Communication Scales (ESCS)

We used three tasks from the Early Social Communication Scales (Mundy, 2003) to assess infants' social attention and social cognitive skills during structured observations with the Assessor. The tasks were administered while the infant sat on the parent's lap across a table, with the assessor sitting opposite the infant.

Object spectacle task: The Assessor activated a mechanical wind-up toy in front of the infant, but ensuring it was out of reach. The Assessor remained silent, but if the child initiated a communicative bid (e.g. alternated her gaze between researcher and toy), the researcher provided a natural but brief response (e.g. smiled and nodded). If the infant tried to obtain the toy, the Assessor moved the toy within the infants' reach. If the toy ceased but the child had not made a bid for it, the experimenter placed the toy within the infant's reach and allowed her to play with it for 10 sec. The task was repeated six times in total, with the first and the last three presentations interspersed between other tasks. The outcomes of interest were Initiating Joint Attention and Initiating Behavioural Requests.

Gaze following task: The Assessor attracted the infants' attention. When the infant was watching the Assessor's face, the Assessor turned his head and pointed to one of four posters located either on the left side, or left behind the infant, or on the rift side, or right behind the infant. While looking and pointing at the poster, the Assessor called the child's name three times, each time more emphatically. The Assessor's turn and pointing to the poster lasted at least 6 seconds. After this, the Assessor attracted the child's attention back. The Assessor initiated another trial when the infant was looking at the Assessor's face. The Assessor turned and pointed to each one of the four posters. The order of direction of turns was pseudo-randomised. The task assesses infants' ability to follow another's person gaze towards a target.

Book presentation task: The Assessor presented a picture book within infant's reach. The Assessor asked the child "What do you see?" and allowed the child 20 seconds to examine the book. If the child pointed to pictures in the book, the Assessor responded briefly (e.g. "Yes, I see"; "That's nice"). After the initial 20 seconds, the Assessor started pointing to pictures in the book, regardless of whether the infant had spontaneously pointed at them or

not. Each pointing gesture was maintained for at least 3 seconds, avoiding tapping or touching the picture while pointing. The Assessor also called the child's name while pointing to pictures on the left side of the book, then point to a new picture on the right side on the same page. After these two pointing gestures, the Assessor repeated this procedure two times, turning onto a new page in the book. This task assessed infants' ability to initiate joint attention bids (e.g. spontaneously point to pictures, alternating gaze between experimenter and pictures) and their ability to respond to joint attention (follow the experimenter's pointing gesture).

Main outcomes

Responding to Joint Attention (RJA): This behaviour category refers to infants' ability to follow the Assessor's line of regard and pointing gestures. In particular, it included: instances of *following the proximal pointing* when the Assessor pointed to pictures in the book during the Book Presentation task*; following the line of regard* when the Assessor turned and pointed to different posters during the Gaze Following task. Since the Assessor pointed or turned to a single target in each trial being administered, in data analyses we considered the proportions of trials whereby the infant correctly followed the target of the trial.

Initiating Joint Attention (IJA): This behaviours category refers to infants' use of eye contact, pointing, and showing objects to initiate bouts of shared attention to objects or events together with the Assessor or the Parent. In particular, this category included: instances of *eye contact* with the Assessor while the infant manipulated an inactive mechanical toy in the Object Spectacle task; instances of *alternate looks* between the active object and the Assessor in the Object Spectacle task; instances of *pointing* to an active toy in the Object Spectacle task, pointing to the posters in the Gaze Following task, or to pictures in the book in the Book presentation task; instances of *showing* an object in the Object Spectacle task, whereby the infant held the toy and raised to the Assessor's (or the parent's) line of view. Since infants could display different instances of these behaviours, in data analyses we considered the *rate* of this category of behaviours, calculated as the number of these behaviours displayed divided by the trial duration (in minutes).

Coding and Reliability

A rater blind to infants' group allocation coded the recorded tasks continuously, indicating any instance of the RJA and IJA behaviours. A second rater coded all the tasks to check for inter-rater agreement. This was adequate in the *Gaze Following Task,* with percentage of agreement in coding infants' direction of gaze being 87%, $\kappa = 0.72$.

Agreement was excellent in coding infants' behaviour during the *Object Spectacle Task* (range from 90% to 100% of agreements, $\kappa = 0.84$ to $\kappa = 1.00$) and during the *Book Presentation task* (92% of agreements, $\kappa = 0.84$).

Lab-Tab Tasks: Attractive Toy Placed in a Box

The Assessor sat 90 degrees from the infant holding some papers to read. The experimenter instructed the parent saying: "In this task we are interested to see how children react when the toy they are playing with is taken away for 30 seconds at a time. [name of child] is likely to show some signs of frustration during this task, but I will only take the toy away on three occasions and he/she will have a chance to play with it in between. Please do not help him/her retrieve the toy. If he/she seeks your involvement, you may say "yes, the toy is in the box'. If he/she seeks your comfort, by all means comfort him/her. To avoid distraction, I will not talk to [name of child] during this task. If you feel uncomfortable continuing with this task at any point, please tell me and I will stop the task immediately". The Assessor then displayed an attractive toy (a toy phone that emitted lights and sounds) and showed the infant how it could be played with, and then placed it within the child's reach. After the child had played with it for 30 seconds, the Assessor took the toy and placed it in the box, closing the lid and leaving it there for 30 seconds. During the latter period, the experimenter pretended to be reading from his papers. The toy was then returned to the infant, and the procedure repeated two more times, for a total of three trials. The task assessed infants' orienting, social referencing, and behaviour control.

Coding and Reliability

A coder blind to infants' group allocation divided each of the three 30 s trials into intervals of 5 s. Within each of these intervals, the coder rated infants' peak display of: *struggle* from 0 (no struggle) to 3 (continuous movements of moderate high intensity to get the toy); *intensity of facial anger* from 0 (no facial region showing codable anger) to 3 (codable signs of anger in all three facial regions); *intensity of distress vocalisations* from 0 (no vocalisations indicating distress) to 4 (full intensity cry or scream). Parental interference was also coded during each interval from 0 (no interference) to 1 (parent actively trying to sooth or calm the infant).

A second scorer coded a random sample (n= 7) of the recorded tasks (approximately a third of all those recorded) to check coding reliability. Inter-rater agreement was satisfactory ranging from 95% for *intensity of struggle*, to 98% for *distress vocalisation,* and $\kappa = 0.92$, $\kappa =$

0.88, $\kappa = 0.97$, and $\kappa = 0.85$ for *intensity of struggle, intensity of facial anger, intensity of distress vocalisation,* and *parental interference* respectively.

Outcome in Analyses

The main outcome was an average of infants' intensity of struggle, intensity of facial anger, and intensity of distress vocalisation across each trial. Ratings of parental interference (e.g. parents trying to vocally sooth the infant) varied across groups, being 0.14 and 0.06 at pre-, and 0.10 and 0.05 at post-test for control and treated infants respectively: for this reason, ratings of infants' anger in each of the three sessions were weighted by the parental interference average score in the same session.

Mullen Scales of Early Learning

The Mullen scales represent a validated assessment of cognitive and motor abilities suitable for infants from birth to 68 months of age (Mullen, 1995). The assessment involves presenting age-appropriate tasks to the infant (e.g. invite the infant to roll a ball) using test material similar to ordinary toys (e.g. toy cars, balls, etc.). The scales provide standardised scores in different areas: gross and fine motor abilities, visual reception, expressive and receptive language. Furthermore, these scores can be used to calculate an Early Learning composite score, thus providing a validated and standardised test of general cognitive abilities.

References

Gagne, J. R., Van Hulle, C. A., Aksan, N., Essex, M. J., & Goldsmith, H. H. (2011). Deriving Childhood Temperament Measures from Emotion-Eliciting Behavioral Episodes: Scale Construction and Initial Validation. *Psychological Assessment, 23(2),* 337–353. https://doi.org/10.1037/a0021746

Mullen, E. M. (1995). *Mullen Scales of Early Learning: AGS Edition. American Guidance Services, Inc.* https://doi.org/10.1002/9780470373699.speced1402

Mundy, P. (2003). *Manual for early social communication scales (ESCS)*. Coral Gables, FL.

Planalp, E. M., Hulle, C. Van, Gagne, J. R., & Hill Goldsmith, H. (2017). The infant version of the laboratory temperament assessment battery (Lab-TAB): Measurement

properties and implications for concepts of temperament. *Frontiers in Psychology, 8,* 846. https://doi.org/10.3389/fpsyg.2017.00846

Putnam, S. P., Helbig, A. L., Gartstein, M. A., Rothbart, M. K., & Leerkes, E. (2014). Development and assessment of short and very short forms of the infant behavior questionnaire-revised. *Journal of Personality Assessment, 96(4)*, 445–458. https://doi.org/10.1080/00223891.2013.841171

Section 5
Introduction to Bayesian Analyses

Bayesian statistics is an approach to data analyses and inference that takes its name from Bayes' rule (Bayes & Prince, 1763), which provides a formal method for combining prior information (i.e. information gathered *before* collecting new data) with evidence from observed data. Bayesian statistics is not a novel approach, but it has gained traction in recent decades thanks to advances in computation, which have increased ability to carry out the calculations and stochastic simulations integral to the approach.

One way to highlight the characteristics of Bayesian statistics is to contrast it with the traditional statistical approach, which is often dubbed *frequentist.* The key difference between the frequentist statistical approach and the Bayesian one lies in the way these approaches define population parameters, such as differences in means. The frequentist approach assumes parameters (e.g. difference in means between two groups) are unknown but fixed quantities: it is possible to estimate the "true" value of the parameter through a hypothetical resampling of the data whereby one can imagine the same study being repeated over several occasions. In the frequentist approach the mean difference observed in a study is thus considered one of the possible values from the hypothetical resampling of the data. In the traditional null-hypothesis procedure, this value is compared to the sampling distribution that would be expected in case there were no differences between groups. The results do not inform about the probability of the parameter of interest, but rather the probability of the parameter taking the observed value, or a more extreme one, if the null hypothesis were true. Null hypothesis testing has been criticised for leading to publication bias, and emphasis has now shifted to reporting estimates of uncertainty such as confidence intervals. However, while confidence intervals are often erroneously interpreted as the range of parameter values that are probable, their interpretation relies on the same counterfactual scenario as the null hypothesis, as they represent ranges of values that would not be rejected with a certain probability. Thus, the traditional statistical approach cannot provide answers to questions concerning whether the difference between groups falls within a certain range, or the probability of this difference being a specific value of interest.

These questions can be answered instead by Bayesian analysis. Bayesian analysis assumes that parameters such as differences between means are uncertain, and therefore can be described by a probability distribution. Thus, the approach can answer questions about the probability of a difference being above a certain value, or within a certain range. Indeed, Bayesian analyses report *Uncertainty Intervals*, which represent the range of parameters that

can be observed with a certain probability. Conventionally, researchers that use this approach report ranges with 89% probability, rather than 95%. The assumption that parameters have a probability distribution has another important consequence: the parameter distribution can be updated by new data being collected.

Indeed, the fundamental principle of Bayesian analysis is that *a-priori* assumptions about the distribution of a parameter are updated considering new data (Kruschke & Liddell, 2018; van de Schoot et al., 2014). A-priori assumptions about a parameter are formally expressed by a *prior* distribution: This distribution represents a plausible model of how the data are expected to be distributed. This model is conceived before new information is collected and analysed. Prior distributions can be based on theoretical assumptions or previous research (e.g. meta-analyses).

Prior distributions are updated by new evidence becoming available or being collected (e.g. in a novel experiment): this process allows to revise a-priori models to create a *posterior* distribution that better accounts for the new data collected. The posterior distribution is thus a new, updated, and credible distribution of the parameters of interest.

Bayesian analysis has thus been described as a process that "takes a question in the form of a model and uses logic to produce an answer in the form of probability distributions" (McElreath, 2018: Page 10). Some of the attractive features of this approach consist in the ability to include previous knowledge in the analyses, and in the fact this information is formally and transparently formalised in a *prior* distribution. Furthermore, every piece of information, no matter how small, can be meaningfully used to update prior models: inference in Bayesian analyses is *informative* and *valid* regardless of the sample size. Inference in fact is not justified on methods that assume asymptotic behaviour of samples akin to that of large size samples, as in the frequentist approach. Overall, Bayesian analyses provide meaningful methods to estimate plausible parameters and quantify uncertainty around these estimations regardless of sample size and without invoking assumptions relying on hypothetical repeated data collections. The references cited below provide accessible introductions to Bayesian statistics.

References

Bayes, T., & Price, R. (1763). An essay towards solving a problem in the doctrine of chance. By the late Rev. Mr. Bayes, communicated by Mr. Price, in a letter to John Canton,

M. A. and F. R. S. *Philosophical Transactions of the Royal Society of London, 53*, 370–418. doi:10.1098/rstl.1763.0053

Kruschke, J. K., & Liddell, T. M. (2018). Bayesian data analysis for newcomers. *Psychometric Bulletin & Review, 25(1)*, 155–177. https://doi.org/10.3758/s13423-017-1272-1

McElreath, R. (2018). *Statistical rethinking: A Bayesian course with examples in R and Stan* (Second Edi). Abingdon, UK: CRC Press. https://doi.org/10.1201/9781315372495

van de Schoot, R., Kaplan, D., Denissen, J., Asendorpf, J.B., Neyer, F.J., & van Aken M.A.G. (2014). A gentle introduction to Bayesian analysis: applications to developmental research. *Child Development, 85(3)*, 842-860. doi: 10.1111/cdev.12169.

Section 6
Summary Scores Used in Analyses

We used Bayesian analysis to estimate how the treatment related to changes across pre- and post-assessments. To this aim, for each outcome we calculated *difference scores* by subtracting infants' scores in the pre-test from the post-test scores. To ensure these scores were easier to interpret, we reversed the difference scores of those outcomes whereby lower scores indicated better performance, and particularly:

(a) The number of looks to habituation in the *Visual Habituation task*, whereby lower numbers indicated fewer looks to reach habituation criteria, had been transformed using their reciprocal to allow for asymmetrical distributions.

(b) Disengagement latencies in the *Gap-Overlap task*, whereby lower raw scores indicated quicker turns to the target, had been inverse-transformed;

(c) Attention capture in the *Information Density task* had been inverse-transformed to account to the fact that lower raw scores indicated more attention to the slower stimuli presentation, compared to the attention-eliciting fast one.

(d) Average protest in the *Lab-TAB Toy in a Box task*, whereby lower raw scores indicate milder display of protest, had been inverse-transformed.

After these scores were transformed, all difference scores were thus coded in such a way that *higher scores* indicated *better performance at post-test.* The difference scores described were then standardised into *d* scores by calculating each participant's difference from the sample mean and dividing it by the sample standard deviations. Thus, $d = 0$ indicated no differences in the outcome between pre- and post-test, whereas $d = 1$ indicated an improvement from pre to post-test of 1 *SD* unit, and so on.

The Bayesian regression analyses allowed to further estimate the average differences in *d* scores between treated and controls: we indicate these as $\hat{d}_T$ scores. Thus, $\hat{d}_T$ scores represent the expected average post-test improvement of treated children compared to controls, expressed in *SD* units: e.g. a positive $\hat{d}_T = 1$ estimated that the treated displayed a 1 *SD* gain in post-test scores, on average, compared to the controls.

Section 7
Bayesian Regression Models

Two key elements are pivotal in Bayesian analysis: a *prior* distribution of each parameter being estimated, and a *likelihood function*.

A prior distribution formally describes the expected distribution of parameters before considering the data at hand. As we will detail in this section, we assumed there were no differences between treated and control in the outcomes: in this way, the most plausible model *before* collecting our data considered that the treatment would have had no effect in improving outcomes.

The *likelihood function* represents a distribution function assigned to an outcome. In a similar manner to standard regression analyses, predictors are included in these analyses by expressing the outcome as a function of the predictor variables: We used linear regression models whereby the outcome was the result of a linear combination of predictors (e.g. group allocation).

The *prior* distribution and the *likelihood function* are combined using Bayes' rule to generate a *posterior* updated probability distribution of the parameters of interest. The posterior distribution is then used to simulate samples that allow to estimate the most plausible parameter values and what range of values covers the most plausible intervals (Kruschke & Liddell, 2018). We used samples from the posterior distributions to answer questions about *point estimates* (i.e. estimated difference between treated and controls), *defined probability mass* (i.e. the interval of parameter values with higher probability) and *defined boundaries* (i.e. how much posterior probability of the difference between groups lied above 0, or else, indicated improvements for the treated). In line with other authors who have used this approach, we reported *89% Uncertainty Intervals* (UI) for each parameter estimated.

We ran the analyses in R (R Development CoreTeam, 2020) using Hamiltonian Monte Carlo (HMC) sampling implemented in the "ulam" command from the package "Rethinking" (McElreath, 2018). In estimating the posterior distribution, we used four chains, each with a sample size of 20000 draws following a warm-up of 10000 draws. Convergence of models was checked using graphical methods (inspection of trace plots and trank plots of the estimation process): the number of draws used displayed convergence in all the models we tested.

Models Tested and Model Selection

We tested three regression models in sequence. In Model 1, the *d* scores (see Supplementary Material, Section 4) were expressed as a function of the treatment/control allocation. Model 2 included an effect of age independent of the treatment effect: namely, the *d* scores were expressed as a linear function of treatment allocation *and* infants' age. Model 3 considered an interaction between age and treatment: the assumption of the model was thus that treatment effects were conditional on and could vary according to infants' age. Results of model comparisons for each outcome are presented in Table 5 of the manuscript. Prior distributions of each parameters in the model are formally presented in what follows.

Model 1: Treatment Effect

The model expressed the distribution of the outcome *d* as a linear function of the treatment/control allocation. A linear regression function with a categorical variable like treatment/control can specify the outcome as a function of an *intercept,* representing the expected average of the outcome when all predictors are absent (e.g. treatment is absent), and a *slope,* representing the difference in the outcome expected in the presence of the predictor. However, by specifying an additional parameter associated with treatment (the *slope*), the estimates of treatment effects have more uncertainty attached, since these are the result of two parameters (*intercept* and *slope*) with two prior distributions. The other drawback of this approach is also evident when interaction terms between predictors are included in models (as in our Model 3 further below): in this case, the introduction of a further parameter indicating the interaction between predictors (e.g. treatment and age) would further inflate the uncertainty associated with the predictor.

In order to avoid these issues, we did not specify a prior distribution for the treatment effect (i.e. the treatment slope), but rather estimated the treatment effect using the indexing approach described by McElrath (2018). In this approach, the treatment and the control group are indexed by a number that represents the different categories of the variable: the model then specifies that the parameter in the model (i.e. the intercept) can assume different values for the indexed variables. The model will thus estimate an intercept for each indexed category of the categorical variable, i.e. an intercept for the treated and one for the controls. However, the same prior distribution would be assigned to these parameters, which represents the expectation that all the categories are the same. Hence, no differences in the outcomes of the treated were expected in comparison to the controls.

Thus, the model was formally expressed by:

$d_i \sim$ Normal (μ_i, σ)

$\mu_i = \alpha_{Treatment[i]}$

$\alpha_j \sim$ Normal(0,1) for j = 1 (Treatment), 2 (Control)

$\sigma \sim$ Exp(1)

In the model, d_i represents the standardised difference score for the *i*th participant. The first line thus indicates that standardised difference scores are expected to approximate a normal distribution with mean μ and standard deviation σ. The mean score of the *i*th participant, μ_i, is expressed as a function of an intercept α, which differs according to which of the two categories *j* of treatment participant *i* was allocated. The two categories were indexed as 1=Treatment, and 2=Control. We emphasise that the indexing integers 1 and 2 do not imply an order but are just used to describe different categories of the variable.

The intercept α_j is expected to be distributed normally with mean 0 and standard deviation 1. Namely, this prior distribution assumes that both intercepts for the treated (α_1) and the controls (α_2) are normally distributed with mean 0 and standard deviation equal 1. Once again, we emphasise that, prior to the data, this model expected treated and controls to display the same outcome distribution. Finally, the standard deviation σ of the standardised difference scores *d* are expected to follow an exponential distribution with parameter 1, which constrains the standard deviation to be positive. While d_i is the outcome, μ_i, α_1, α_2 and σ are model parameters for which prior distributions are specified.

Model 2: Treatment Effect and Age Effect

The second model added a parameter to estimate changes in *d* scores associated with different ages at which infants were tested. As indicated in the manuscript, this was justified by the fact that infants had been tested at different ages at post-test ranging from 12 to 16 months, and age varied between treated and controls.

The age variable considered was infants' age at post-test, centred at 12 months, so that a score of 1 indicated age 13 months (i.e. 1 month after 12 months). Infant's age was considered since conception (estimated based on the infants' expected date of birth) in order to control for infants' prematurity.

We expected a small increase in *d* scores associated with age increasing by 1 month, and thus assumed an increase equivalent to 0.10 *SD* units, with a standard deviation of 0.35 *SD* units. Thus, Model 2 was formally expressed as:

$d_i \sim$ Normal (μ_i, σ)

$\mu_i = \alpha_{Treatment[i]} + \beta \, Age_i$

$\alpha_j \sim Normal(0,1)$ for j = 1 (Treatment), 2 (Control)

$\beta \sim Normal(0.10, 0.35)$

$\sigma \sim Exp(1)$

Compared to Model 1, this model included a further parameter β representing the difference in d_i associated with 1 month increase in infants' age. The prior distribution of this parameter was assumed to approximate a normal distribution with mean 0.10 and SD 0.35. Thus, it was expected that from one month to another, 95% of d_i scores could vary between a -0.60 SD units decrease and a 0.80 SD units increase.

Model 3: Interaction between Treatment and Age

The third model assumed an interaction between infant's age and group allocation. The model thus assumed that treatment effects were moderated by the infants' age. Namely, treatment effects varied depending on the age of testing.

The model is formally expressed as:

$d_i \sim Normal(\mu_i, \sigma)$

$\mu_i = \alpha_{Treatment[i]} + \beta \, Age_{Treatment[i]}$

$\alpha_j \sim Normal(0,1)$ for j = 1 (Treatment), 2 (Control)

$\beta_j \sim Normal(0.10, 0.35)$ for j = 1 (Treatment), 2 (Control)

$\sigma \sim Exp(1)$

Comparing Model 3 to Model 2 reveals that the parameter representing age effects β is indexed by j, representing treatment or control allocation. Thus, the model estimated two parameters, β_1 and β_2 representing how much d_i changed with infants' age for the treated and the controls respectively. In the same manner as in Model 1, the prior specifies that these two parameters are expected to show the same distributions, representing an expectation of the two groups being similar.

Models Comparisons and Treatment/Control Contrast Calculations

For each outcome of interest, the three models described were compared using the Widely Applicable Information Criterion (WAIC), developed by Watanabe (2010). The WAIC allows to compare models according to their expected predictive value while

accounting for overfitting. Lower WAIC scores indicate increased predictive value. We thus selected the models with the lower WAIC value: estimates and predictions from the models selected have been reported in the manuscript. In Table 5 of the manuscropt we report the results of model comparisons for each outcome.

Estimates of $\hat{d}_T$

Contrasts between treatment and controls were estimated by sampling from the posterior distributions of the model selected for each outcome. Samples from the posterior distributions allow to control for uncertainty about estimated parameters. The size of these samples from the posterior distribution was $n = 10{,}000$. From these samples, we were able to estimate the average differences in *d* scores between treated and controls, as well as the 89% *Uncertainty Intervals* of these differences, and the probability of this difference being above 0, i.e. favouring the treated. The distributions of the *d* scores from these samples are reported in Section 6 of Supplementary Materials.

As described in Section 4 of Supplementary Material, we dubbed the average differences of *d* scores between treated and controls as $\hat{d}_T$. In cases the models selected indicated treatment effects were moderated by age, we estimated the $\hat{d}_T$ at age 13 months. This age was chosen as we had planned for the intervention to start at 12 months and to be concluded around age 13 months. The results of these analyses are reported in Table 6 and 7 of the manuscript. In Figure 2 and 3 of the manuscript we report the estimated distribution of the $\hat{d}_T$ scores by outcome.

References

Kruschke, J. K., & Liddell, T. M. (2018). Bayesian data analysis for newcomers. *Psychometric Bulletin & Review, 25(1)*, 155–177. https://doi.org/10.3758/s13423-017-1272-1

McElreath, R. (2018). *Statistical rethinking: A Bayesian Course with Examples in R and Stan (Second Edition)*. Abingdon, UK: CRC Press. https://doi.org/10.1201/9781315372495

R Development Core Team (2020). *R: A Language and Environment for Statistical Computing. R Foundation for Statistical Computing*. Vienna, Austria. https://doi.org/10.1007/978-3-540-74686-7

Watanabe, S. (2010). Asymptotic Equivalence of Bayes Cross Validation and Widely Applicable Information Criterion in Singular Learning Theory. *Journal of Machine Learning Research, 11*, 3571–3594.

Section 8
Distributions of *d* scores by outcome

In this section we report the estimated *d* scores from the posterior distributions by outcomes. The *d* scores represented standardised differences between post- and pre-test. Positive scores indicated better performance at post-test (see Section 5 of Supplementary Material, and Methods in the manuscript). In the following figures, we report the estimated distributions of these *d* scores for the treated (colour-coded in red) and the controls (colour-coded in blue). These figures are useful in emphasising whether groups reported *d* scores above 0, i.e. indicating improvement at post-test, as well as differences between treated and controls (represented in Figure 2 and 3 of the manuscript). The point equivalent to $d = 0$ (i.e. no change between pre- and post-test) is indicated by a dotted line. The vertical axis in the figures represent the density of the estimated probability of the *d* scores on the horizontal axis.

Sustained Attention: Peak Look

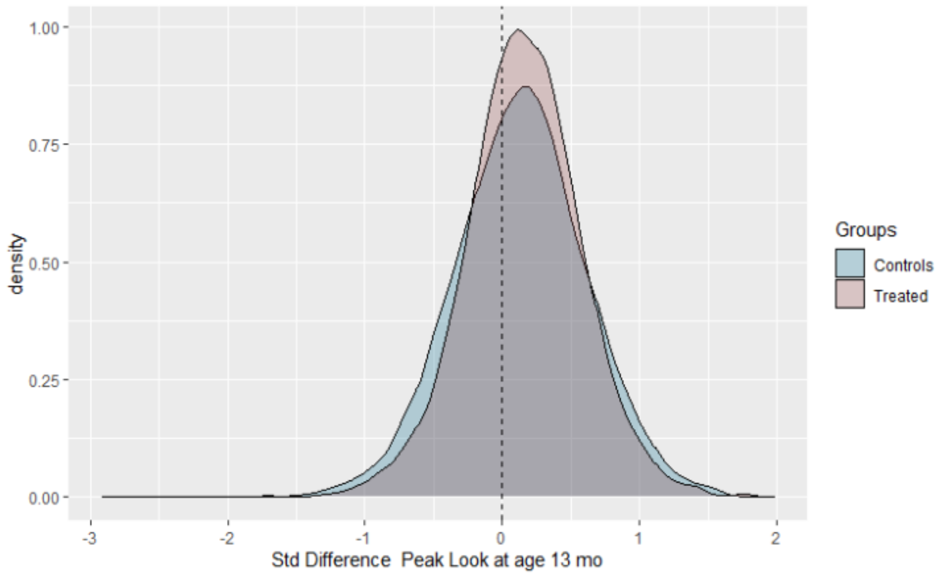

Sustained Attention: Reciprocal Looks to Habituation

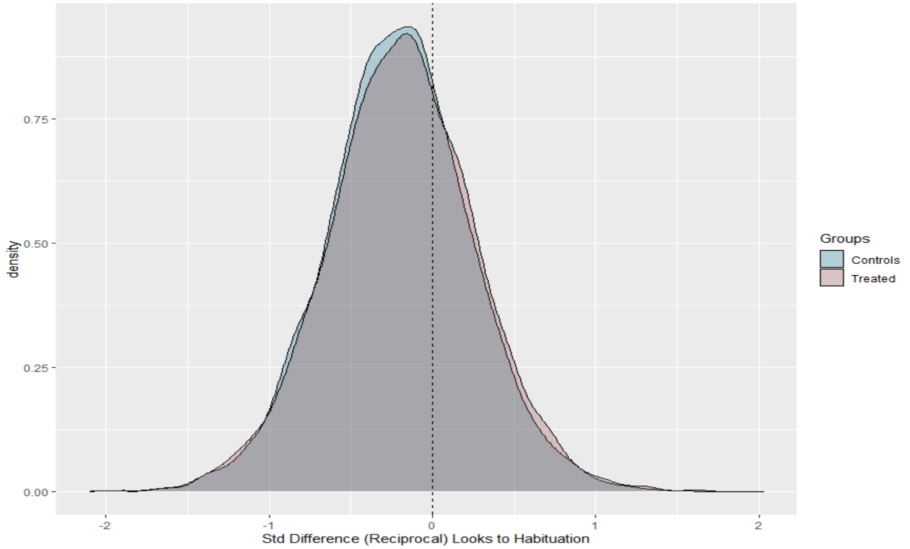

Visual Memory: Novelty Preference

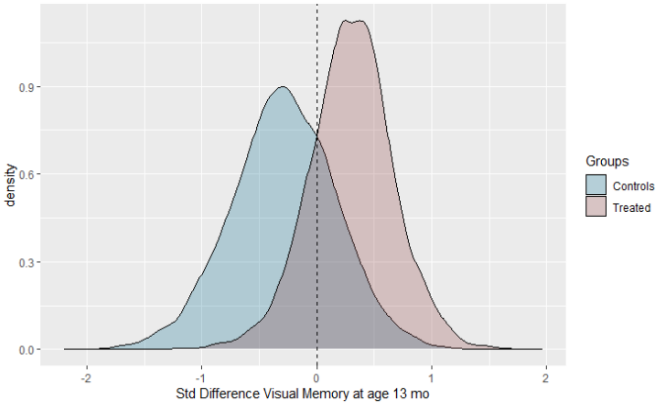

Gap-Overlap: Inverse Disengagement

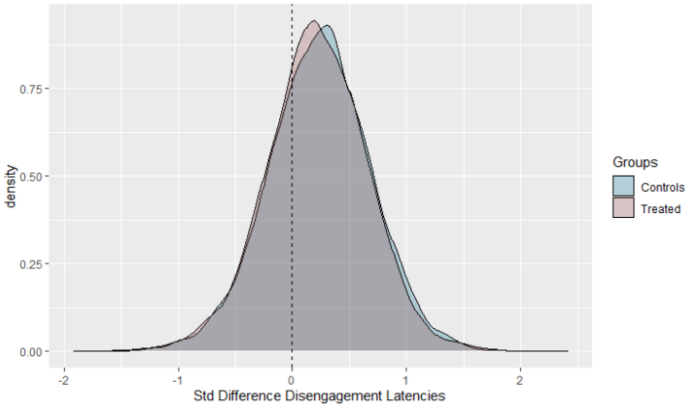

Information Density: Inverse Attention Capture

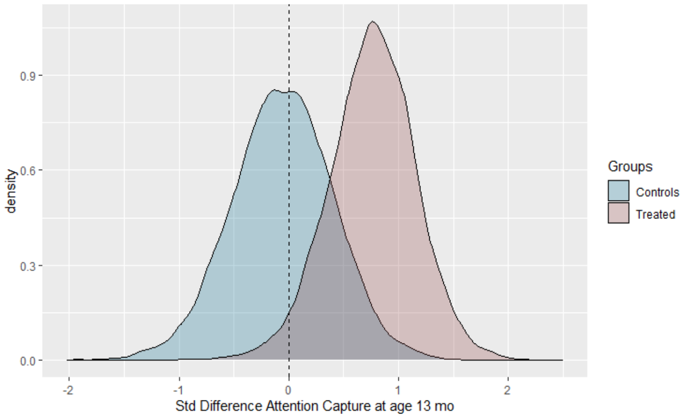

LabTab Orienting: Facial Interest

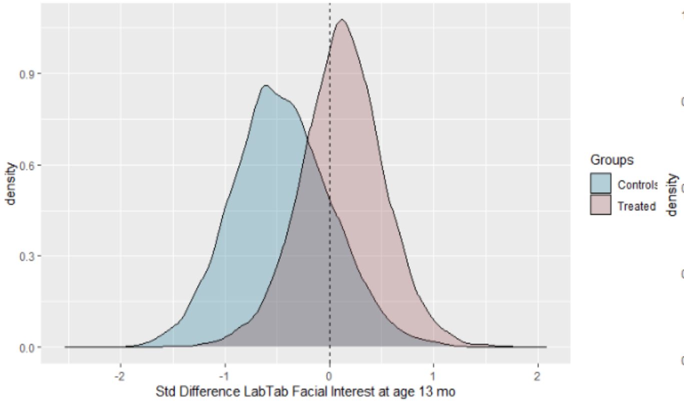

Free Play: Looking at Objects

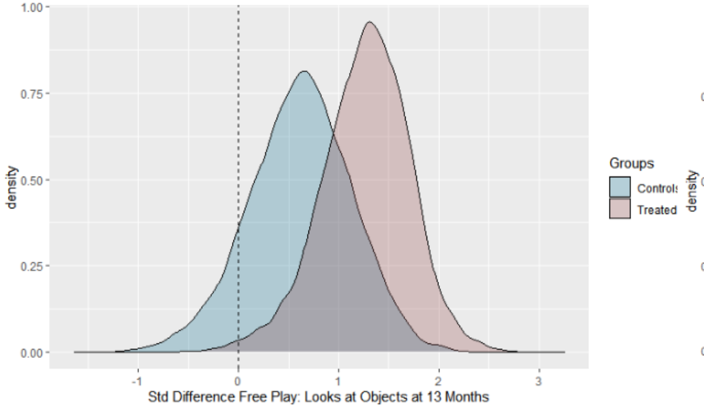

IBQ: Effortful Control

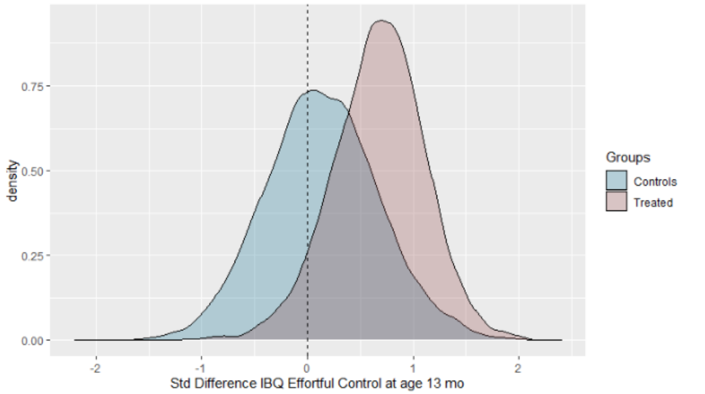

ESCS: Initiating Joint Attention

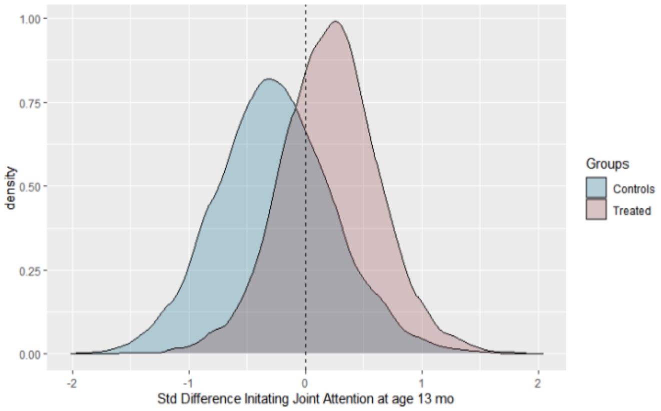

ESCS: Responding to Joint Attention

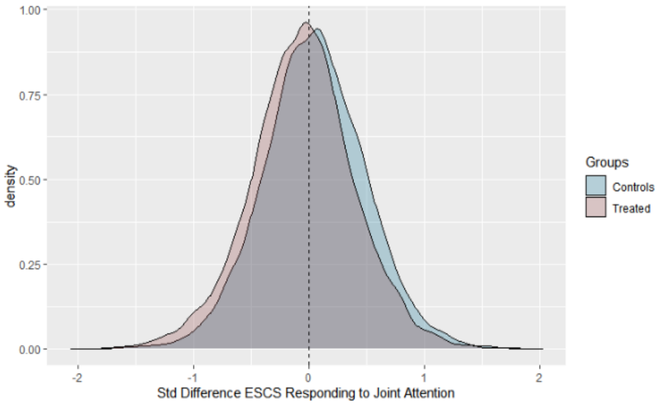

LabTab Toy in Box: Inverse Intensity of Protest

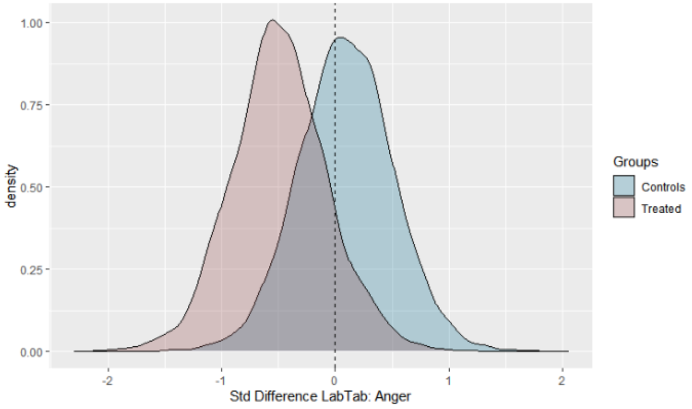

Supplement: S1 File — (PDF) [file pone.0273767.s002.pdf]
