# Supplementary material for: Attention and social communication skills of very preterm infants after training attention control: Bayesian analyses of a feasibility study
Source: PLoS One. 2022 Sep 22;17(9):e0273767. doi: 10.1371/journal.pone.0273767 (PMC9499320; doi:10.1371/journal.pone.0273767)
Supplement: S2 File — (DOCX) [file pone.0273767.s003.docx]

## A feasibility study of the Attention Control Training (ACT) intervention amongst very preterm (VP) infants.

## Introduction

The incidence of premature births in high income countries has increased in recent decades(1,2), and more infants born preterm survive. In England and Wales(3) and Northern Ireland(4) approximately 7% of all live births take place prematurely (less than 37 weeks’ gestation). Premature birth is associated with increased risk for intellectual problems and lower educational achievement(5,6). Risk for these unfavourable outcomes increases amongst infants of lower gestational age. Children born between 28 and 31 weeks are defined as very preterm (henceforth: VP) and are at risk of persistent learning difficulties(6,7) and problem behaviour such as attention deficit disorder(8) even in absence of major health complications.

Several studies(9-13) indicate that the basis of learning and behavioural problems of VP children lies in impaired ability to regulate the acquisition and retention of information during infancy. One of the key processes that are disrupted in VP infants is attention control, which indicates infants’ ability to select actively what to pay attention to and what to ignore(14): active control over the flow of information attended enables infants to focus on task-relevant information. In typically developing infants, attention control starts to emerge around 1 year of age and develops throughout early childhood(15). Attention control may be impaired in VP infants because prematurity is associated with disruptions of brain development, which may ultimately affect neural plasticity(16).

VP infants display problems in attention control from an early age(10,17), and these problems persist over time(11,18,19). Longitudinal studies show that problems in attention abilities mediate the association between prematurity and cognitive difficulties(10, 20, 21). Attention control problems in infancy may thus cause a cascade of effects that disrupts the development of other abilities(12), such as Executive Functions (EFs), and these in turn may affect children’s learning(22) and behaviour regulation(23). These links from prematurity to early attention difficulties, poorer EFs(24,25), learning and self-regulation problems(26,27), emphasise the importance of intervening at an early age, providing infants with remedial processes at a time of greater plasticity of brain networks and before deficits are entrenched. Early interventions targeted at key early skills can help infants attain the necessary building blocks for later attainments.

A new intervention to target infants’ attention control has been developed(28,29), the Attention Control Training (ACT). This intervention uses computer interfaces that monitor infants’ direction of gaze through eye-tracking: this allows the computer to provide interactive presentations whereby visual stimuli on the screen change and vary in response to infants’ gaze direction. Using interactive presentations, infants are presented with adaptive challenges, i.e. challenges appropriate for the initial skill-set of each individual child. The use of computer interfaces also facilitates the ACT delivery with fidelity across centres and with reduced personnel costs.

The ACT has been trialled with normally-developing children and has provided evidence of transfer of effects to attention and memory skills (28, 29), thus demonstrating generalizability. Adapting this intervention for VP infants meets the need for targeted early interventions(30-32). Furthermore, the coherent theoretical background of this intervention and its specific focus meet the need for research on the key mechanisms of early intervention amongst preterm infants, a need highlighted by a recent Cochrane review(33). However, before the intervention can be tested with VP infants, a series of processes need to be investigated to underpin its implementation in a full-scale randomised trial. These processes are the subject of this project.

## Aims and Objectives

We will conduct a feasibility study to test processes we plan to use in a full-scale randomised trial of the effectiveness of the ACT intervention with VP infants. These processes will include adapting the ACT procedure to the VP population, testing the quality of the information collected during training and assessments with the VP population, investigating acceptability of a RCT procedure and the ACT schedule by parents of VP infants.

The aims of this project are to:

(a) Investigate VP infants’ engagement with the ACT procedure;

(b) Assess the quality of data collected during the training and assessments used, and compare them to similar data obtained from typically developing infants who completed studies conducted by collaborators.

(c) Investigate retention into the programme.

(d) Investigate infants’ performance in outcome measures and assess trends indicating differences in performance between treated and controls.

This protocol presents the procedures and methods we will use to fulfil these aims. These will involve the running of a feasibility RCT whereby 20 infants will be recruited and randomly allocated to the intervention (the ACT training) or control procedure (regular visits to watch standard cartoons). This feasibility study, its aims and its procedures, have been developed in collaboration and consultation with the charity TinyLife, which is a co-applicant in the grant.

## Methods

### Trial design

The study involves testing the feasibility of a randomised trial in which eligible infants are allocated to one of two groups when they are aged 12 months (age corrected for gestational age, corresponding to approximately 14 months of age from the actual date of birth):

(1) The ACT training programme delivered across consecutive weekly sessions for a month (n=10);

(2) Control (n=10), whereby infants will be shown age-appropriate cartoons over the same number of weekly sessions and for a corresponding amount of time as infants in the ACT intervention.

The sample size has been determined using power calculations based on a confidence interval approach (34). While the effect size reported in a previous study conducted with typically developing infants was 0.69 (29), we considered this effect may be substantially smaller amongst VP infants. Using a Cochrane review of interventions involving infancy outcomes of preterm infants (33), we estimated this could be close to 0.40. Using the formulae reported by Cocks and Torgerson (34), the sample size required to run a pilot with 80% power to detect an effect size equal to 0.40, and considering alpha = .05, would be 18 participants. We will recruit 20 infants in order to allow 10% drop out rate from the study.

### Participants

Eligible participants are infants born very preterm (less than 32 weeks gestation age) residing in Northern Ireland. Approximately 0.7% of newborns in Northern Ireland are born between 28 to 31 weeks of gestation age(4), thus accounting for over 200 infants every year. Participants will be approximately 1 year old at the start of the intervention, age corrected for prematurity. We will exclude infants with: significant visual and/or hearing disabilities; congenital anomalies that may impact on their cognitive and sensory-motor development; a diagnosis of Cerebral Palsy; are taking part in a trial (or have recently taken part in a trial) which may interfere with this study (e.g. by affecting concentration abilities) or represented a significant burden for the family. We will ask the child’s main caregiver to provide some information about the child and her behaviour. Furthermore, the caregiver that will be present during the sessions will be asked to involve the child in free-play during pre- and post-test assessments. We expect that in most of the cases the main caregiver that will provide information and take part in these free play sessions will be one of the child’s parents (either the mother or the father). However, we will not exclude infants that are being looked after by caregivers other than a parent (i.e. a grandparent).

### Recruitment

The recruitment will take place with the involvement of Co-I David Sweet, consultant neonatologist at the Royal Maternity Hospital in Belfast, and is described in a schematic form in *Figure 1*. Dr Sweet will act as a *co-ordinator* of the study. He will contact paediatricians in the Belfast Trust to let them know about the study and inform them that we would like their collaboration in passing information about the study to families of potentially eligible babies who were born in Belfast.

Paediatricians typically review ex-preterm babies for follow up at around the time infants are 8-9 months (corrected age). Using the admission book from Royal Maternity Hospital, Dr Sweet will be able to identify children that potentially would meet eligibility criteria for this study before they are due their follow-up visit with a paediatrician. We will then prompt the paediatrician providing follow up in advance of the infant’s visit to consider if there are any exclusion criteria. Together with this letter prompting the paediatrician about the study, we will send a study summary, as well as information about the study that the consultant can give to parents, if they are interested in taking part. The information provided will include contact details of the research team, and therefore interested parents will have a chance to contact the researchers to ask questions and receive more information about the study.

We will also collaborate with the charity TinyLife. TinyLife has supported parents of children born preterm for nearly 30 years, and provides a range of support services to families, including baby-massage, counselling, parent groups, etc. Charity workers will identify eligible children, and will pass information to their families. Families will thus have the choice to contact the research team to ask more information about the study. However, since the medical history of these children will not be known by staff at TinyLife, if parents are interested, they will be offered a consultation with Dr Sweet before meeting the research team and receive more information about the study. Dr Sweet will meet the family and, with their consent, check the medical records to ensure the child meets inclusion criteria for the study.

The procedures described above will ensure that the research team will not approach parents directly: the paediatricians and TinyLife will act as gatekeepers, passing information to parents of children that may be eligible. Only when parents have expressed an interest in the study, will they have the opportunity to contact the research team and receive further information.


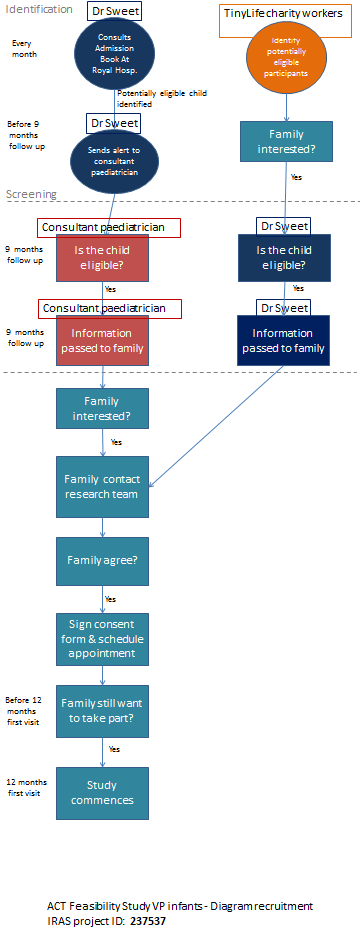


Figure 1: Diagram of recruiting phases

When parents contact the research team to express interest in the study, the RA will provide an overview of the study, allow parents to read the PIS, and also explain the randomisation procedure. At this stage, if the parents are still interested, they will receive the consent form to sign. Parents will be told they will be contacted again when the child is 12 months corrected age. The period between consent and contact preceding scheduling of an appointment will act as a “cool-off” period. If parents are still happy to take part by the time they are contacted again, an appointment will be scheduled. Parents will be reminded that they and their child are expected to take part in five weekly consecutive sessions, but that they can withdraw from the study at any time. They will receive gift vouchers (£50) at the end of the study, as well as compensation for travel expenses incurred in each session attended.

### Randomisation

Randomisation will be computer-generated using ALEA software, with allocation concealed to assessors and parents. The allocated group will be notified automatically to the research assistant responsible for the training.

### Procedure

The first session will include a baseline assessment followed by a first training/control procedure, depending on the child’s random assignment in one group or the other. Weekly session 2, 3, and 4 will involve further administration of training/control procedures. The final session in week 5 will involve collection of outcome measures. All the assessments and the training/control procedures will take place in a dedicated room within the premises of TinyLife. The use of a dedicated room will ensure that infants are tested in controlled conditions whereby interference from noise, changes in light, or other sources of distraction, are minimised. Infant’s parent or parents will be present during the whole of the session and will supervise the infant at all times.

#### Baseline assessment.

We will ask the main caregiver of the child to complete a demographic questionnaire and the short form of the Infant Behaviour Questionnaire (IBQ) (35). These questionnaires will be sent in advance of the appointment, but will be available for parents that may not have had a chance to complete them in advance. Infants will be video-recorded during tasks to allow for further data analyses of behaviour. The researcher will be blind to group allocation of the child during the baseline assessment.

The tasks administered will take approximately 60 minutes in total and are:

(1) Eye-tracker and computer-based cognitive and attention tasks: Using a laptop with an eye-tracker device, we will administer validated measures of sustained attention, attention disengagement, and cognitive control described in more details in the Details of Protocol document.

(2) Naturalistic attention and social attention tasks from the Lab-Tab assessment (Task orientation; Attractive toy placed in box) and from the Early Social Communication Scales

(Gaze following task; Book presentation task).

(3) The Mullen scales, administered by the researcher.

(4) A mother-infant “free-play” session: Mothers will be asked to play with their infant as they normally do, and will have a set of toys available. This free-play session will be used to investigate parenting behaviour and infants’ attention and communication skills in naturalistic conditions (e.g. ability to follow the parent’s direction of gaze).

The tasks will be interspersed by pauses and breaks to allow infants and parents to rest whenever deemed necessary or requested by a parent, or because the infant is sleepy, hungry, etc.

#### Intervention.

Infants in the ACT intervention will watch interactive stimuli presentations that are contingent on infants’ direction of gaze. An eye-tracker will record the infant’s eye movements in real time, while a camera will also record the infant during the training. Collecting video-recording is important for monitoring the quality of data collected (e.g. lack of engagement with tasks by infants) and to potentially complement any loss of data (e.g. when the infant leans too close to the screen). Interactive presentations will provide animations in response to the infant’s gaze in ways that meet pre-specified criteria. We will use the training tasks described in Ballieux et al. (28): (a) Butterfly; (b) Stars; (c) Windows; (d) Suspects. The length of training sessions will vary depending on infants’ engagement with stimuli and time criteria, but are expected to last approximately 30 minutes. However, parents will be reminded that they can stop the sessions whenever they want. The researcher will also assess if the infant is still in calm and alert status before commencing any new task, and will stop the sessions if the infant becomes restless or drowsy.

#### Control.

The control procedure will involve presentation of cartoons on a screen, while infants’ gaze direction will also be recorded using the same eye–tracker and camera. The crucial difference will be that stimuli presented are not interactive, thus do not change contingently with infants’ gaze direction. To ensure presentations in the control procedure are similar in length to those of the intervention group, infants in the control group will be matched infant-by-infant and visit-by-visit with participants in the ACT control, thus ensuring exactly equivalent length of presentations. The cartoons displayed to a control child will follow the same schedule produced by the corresponding yoked child in the intervention group. In this way, the cartoons displayed to both groups will be the same, but the crucial difference will be that the display is generated contingently on infants’ gaze behaviour during treatment, and it is instead generated according to a pre-set schedule for infants in the control group.

#### Final assessment.

The final assessment after the intervention/control procedure will take place in week 5. The same questionnaires and tasks described in the baseline assessment will be used. Parents will be also asked to complete a feedback questionnaire on the study participation. The assessment will take approximately 70 minutes in total.

#### Follow up interview with parents.

**Feedback from families of VP infants that take part in the study will be valuable in designing and planning a future larger trial of the ACT intervention. In this study we ask parents to answer a set of questions about their participation when parents attend the final post-test session. However, a further follow-up semi-structured interview could provide more in-depth, rich information on the facilitators and obstacles to family participation. This information will be valuable in ensuring future trial design address issues that may curtail recruitment, participation, and retention. At the end of the study, parents are asked to sign a consent form to be contacted again to take part in further follow up activities related to this study. We propose to contact all parents that have taken part *and* agreed to be contacted for follow ups, and ask them to take part in a short telephone interview regarding the study. The invitation to this follow up will be provided in an ad-hoc PIS. Parents will then be invited to register their interest in taking part in this follow-up interview by informing a Research team member (e.g. the RA). Once parents have registered their interest, a Research Team member will contact them to agree a date and time for the interview. Parents will be reminded they can always change their mind. On the agreed date, before the interview is conducted by a Research Team member known to the parents, the researcher will first confirm the parent is happy to proceed. At this point, the researcher will inform the parent that we need to register their consent, and to do so the researcher will read a series of statements and ask the parent to confirm agreement with these statements. The researcher will also inform the parent that their consent will be recorded to ensure this is documented, but this recording will be kept separate from the rest of the interview. If the parent agrees to the interview, then the researcher will proceed with the interview following the interview-schedule. The interview will be audio recorded for further analyses. Any information and quotes we will report will be anonymised, reporting only a study number, and we will take care in ensuring that parents and children will be not identifiable from the information we will report.**

### Measures

The primary measures are eye-tracking measures of attention and short-term memory. We will assess these skills using different tasks compared to those involved in the training, thus testing generalizability of results. We will use validated measures of sustained attention, attention disengagement, and cognitive control described in Wass et al. (36). Infants will be assessed using these tasks before randomisation (pre-test) and at the end of the trial in week 5. Secondary outcomes will be naturalistic measures of attention (Lab Tab tasks), social communication and joint attention abilities (Early Social Communication Scales), general measures of cognitive functioning (Mullen scales), parental reports of infants’ temperament and behaviour (Infant Behavior Questionnaire). A secondary outcome of the study includes analyses of the quality of data recorded during assessments and during training sessions. For this purpose, we will use procedures similar to those described by Bailleux et al. (28), which involve comparisons of eye-tracking data quality indexes such as the number of usable fragments and the degree of consistency in the reported position of gaze between recorded samples. We will compare data collected in this pilot trial with data collected from normative samples in previous studies.

### Blinding/Masking

Participant’s group allocation will be known to the research assistant responsible for the training, but will be concealed to the assessor, who will have responsibility for carrying out infants’ assessments at baseline and at end of the trial (week 5). The allocation will also be concealed from the parents by not revealing the nature of the cartoons being displayed to the infant. Parents will be present at all time during testing and training, which poses the risk they could identify the group allocation of their child when watching the cartoons displayed. However, we believe this risk is minimal for a series of reasons:

(a) During training, the infant will be sitting on the parent’s lap, and therefore the parent will not have a direct of view of the infant’s direction of gaze. This will make it difficult for the parent to assess whether the cartoons are interactive or not.

(b) The presentations displayed to the control and intervention group will use the same stimuli, and will be identical. In fact, we will match each child in the control group to a child in the intervention, and the control child will be shown the same exact presentation produced during the trained child’s session.

Indeed, our experience in running these studies is that parents are not aware of whether their child is in the training or control group. However, as part of this feasibility study, we will collect information on this from parents in a questionnaire to be provided at the conclusion of the study.

### Data storage

All the paper data will be stored in a locked bag during transportation between TinyLife and Queen’s University Belfast, and will be stored in a locked cabinet within QUB premises. The electronic and video data will be kept in the encrypted drive of the laptop, copied into a secure network drive within Queen’s, where they will be stored and backed-up, and then securely erased from the laptop drive. Only members of the research team will have access to the data and the video recordings, which will be only used for research purposes. Data and video-recordings will be stored for five years following publication of findings in reports and/or scientific journals, and will successively be destroyed securely.

### Data Analyses

Data analyses will involve comparisons in the outcome measures collected in week 5 between treated and controls using ANCOVA in order to control for baseline measures collected. The analyses will also provide information on the observed effect size, which will be useful in planning future studies.

Other analyses will focus on sample retention across the trial, as well as completion and duration of completed training and assessment tasks by infants. In these analyses, we will also explore family and infant’s characteristics associated with differences in these outcomes, with the view of informing factors that may affect retention and intervention compliance in a larger trial. Further analyses will involve comparisons of indicators of quality of eye-tracker data in this study and data from normative typically developing samples collected across other studies. Indicators used will include the number of usable fragments, or the degree of consistency in the reported position of gaze between recorded samples.

### Ethical Issues

VP infants are potentially vulnerable. The eligibility of babies will be ensured by asking paediatric consultants who are already reviewing these infants to consider if they meet the inclusion criteria, thus safeguarding children that may be more vulnerable or whose conditions may preclude their participation.

It is possible the clinical condition of some infants taking part in the trial may worsen after the initial contact and during study participation e.g. hospitalisation for bronchiolitis: contact with parents after they consented may cause them distress in some circumstances. If such situations arise, we will provide families with information about further support that is available to them (e.g. counselling). We have developed a Distress Protocol that details a course of action in this and similar circumstances.

The tests used in our trial are not designed to provide diagnostic information (e.g. on infants’ cognitive delay). Furthermore, the researchers working on the project are not trained to conduct and deliver diagnostic assessments. The Participant Information Sheets will clarify that the assessments in the study are not designed to provide diagnostic information. Therefore, the PIS will specify that we will not provide information on any individual child’s performance to parents, but we will only provide a report on the aggregate results of the study after its conclusion. However, should our researchers have any concern about the health and wellbeing of a child, we will discuss the concern with the consultant paediatrician involved in this study, and, if considered appropriate by the consultant, we will discuss these concerns with the family or with a relevant authority (e.g. in case of suspected maltreatment).

The anonymity of the information collected from parents and infants will be safeguarded when publishing results by reporting summary statistics and avoiding reporting information that may identify participants. The database used for analyses will only include data on the assessment and outcomes and will not include sensitive information or information that can identify the child or the parents. To this purpose, each child will receive a unique identification code, and personal and sensitive information (e.g. date of birth) will be stored in a separate encrypted file, which will only be accessible by the researcher and the data custodian with credentials. Video recording will also be stored securely in encrypted drives, and labelled only with the study ID of the child. We will ask parents’ consent to be contacted in the future regarding the study and possible follow-up.

**Bibliography**

(1) Beck S, Wojdyla D, Say L, Betran AP, Merialdi M, Requejo JH, et al. The worldwide incidence of preterm birth: a systematic review of maternal mortality and morbidity. Bull World Health Organ 2010;88(1):31-38.

(2) Goldenberg RL, Culhane JF, Iams JD, Romero R. Epidemiology and causes of preterm birth. The Lancet 2008 1/5–11;371(9606):75-84.

(3) Office for National Statistics, Gestation-specific infant mortality, 2013. London, Office for National Statistics, 2015, retrieved on 01/07/2016 at: http://web.ons.gov.uk/ons/dcp171778_419800.pdf

(4) Enquiry by NIMATS via Business Objects (PHA, Health Intelligence), January 2016 (via NICORE, QUB).

(5) Ahlsson F, Kaijser M, Adami J, Lundgren M, Palme M. School Performance After Preterm Birth. Epidemiology 2015 JAN;26(1):106-111.

(6) Allen MC. Neurodevelopmental outcomes of preterm infants. Curr Opin Neurol 2008 APR;21(2):123-128.

(7) Aarnoudse-Moens CSH, Weisglas-Kuperus N, van Goudoever JB, Oosterlaan J. Meta-Analysis of Neurobehavioral Outcomes in Very Preterm and/or Very Low Birth Weight Children. Pediatrics 2009 AUG 2009;124(2):717-728.

(8) Anderson P, Doyle LW, Victorian Infant Collaborative Stu. Neurobehavioral outcomes of school-age children born extremely low birth weight or very preterm in the 1990s. Jama-Journal of the American Medical Association 2003 JUN 25 2003;289(24):3264-3272.

(9) Kaul YF, Rosander K, von Hofsten C, Brodd KS, Holmstrom G, Kaul A, et al. Visual tracking in very preterm infants at 4 mo predicts neurodevelopment at 3 y of age. Pediatr Res 2016 03/30.

(10) Rose SA, Feldman JF, Jankowski JJ, Van Rossem R. A cognitive cascade in infancy: Pathways from prematurity to later mental development. Intelligence 2008 JUL-AUG 2008;36(4):367-378.

(11) Rose SA, Feldman JF, Jankowski JJ. Information processing in toddlers: Continuity from infancy and persistence of preterm deficits. Intelligence 2009 MAY-JUN 2009;37(3):311-320.

(12) Rose SA, Feldman JF, Jankowski JJ. Modeling a cascade of effects: the role of speed and executive functioning in preterm/full-term differences in academic achievement. Developmental Science 2011 SEP 2011;14(5):1161-1175.

(13) Reuner G, Weinschenk A, Pauen S, Pietz J. Cognitive development in 7- to 24-month-old extremely/very-to-moderately/late preterm and full-term born infants: The mediating role of focused attention. Child Neuropsychology 2015 MAY 4 2015;21(3):314-330.

(14) Gazzaley A, Nobre AC. Top-down modulation: bridging selective attention and working memory. Trends Cogn Sci (Regul Ed ) 2012 FEB;16(2):129-135.

(15) Johnson MH, De Haan M. Developmental cognitive neuroscience :an introduction. 3rd ed. Chichester: Wiley-Blackwell; 2011.

(16) Luciana M. Cognitive development in children born preterm: Implications for theories of brain plasticity following early injury. Dev Psychopathol 2003 FAL 2003;15(4):1017-1047.

(17) Sun J, Mohay H, O'Callaghan M. A comparison of executive function in very preterm and term infants at 8 months corrected age. Early Hum Dev 2009 APR 2009;85(4):225-230.

(18) Breeman LD, Jaekel J, Baumann N, Bartmann P, Wolke D. Attention problems in very preterm children from childhood to adulthood: the Bavarian Longitudinal Study. Journal of Child Psychology and Psychiatry 2016 FEB 2016;57(2):132-140.

(19) Pizzo R, Urben S, Van Der Linden M, Borradori-Tolsa C, Freschi M, Forcada-Guex M, et al. Attentional networks efficiency in preterm children. Journal of the International Neuropsychological Society 2010 JAN 2010;16(1):130-137.

(20) Rose SA, Feldman JF, Jankowski JJ, Van Rossem R. Pathways from prematurity and infant abilities to later cognition. Child Dev 2005 NOV-DEC 2005;76(6):1172-1184.

(21) Voigt B, Pietz J, Pauen S, Kliegel M, Reuner G. Cognitive development in very vs. moderately to late preterm and full-term children: Can effortful control account for group differences in toddlerhood? Early Hum Dev 2012 MAY 2012;88(5):307-313.

(22) Thorell LB, Lindqvist S, Nutley SB, Bohlin G, Klingberg T. Training and transfer effects of executive functions in preschool children. Developmental Science 2009 JAN 2009;12(1):106-113.

(23) Volckaert AMS, Noël M. Training executive function in preschoolers reduce externalizing behaviors. Trends in Neuroscience and Education 2015 0;4(1–2):37-47.

(24) Bayless S, Stevenson J. Executive functions in school-age children born very prematurely. Early Hum Dev 2007 APR 2007;83(4):247-254.

(25) Mulder H, Pitchford NJ, Hagger MS, Marlow N. Development of Executive Function and Attention in Preterm Children: A Systematic Review. Dev Neuropsychol 2009 JUL-AUG 2009;34(4):393-421.

(26) Linsell L, Malouf R, Johnson S, Morris J, Kurinczuk JJ, Marlow N. Prognostic Factors for Behavioral Problems and Psychiatric Disorders in Children Born Very Preterm or Very Low Birth Weight: A Systematic Review. Journal of Developmental and Behavioral Pediatrics 2016 JAN 2016;37(1):88-102.

(27) Reijneveld SA, de Kleine MJK, van Baar AL, Kollee LAA, Verhaak CM, Verhulst FC, et al. Behavioural and emotional problems in very preterm and very low birthweight infants at age 5 years. Archives of Disease in Childhood-Fetal and Neonatal Edition 2006 NOV 2006;91(6):F423-F428.

(28) Ballieux H, Wass SV, Tomalski P, Kushnerenko E, Karmiloff-Smith A, Johnson MH, et al. Applying gaze-contingent training within community settings to infants from diverse SES backgrounds. Journal of Applied Developmental Psychology 2016 0;43:8-17.

(29) Wass S, Porayska-Pomsta K, Johnson MH. Training Attentional Control in Infancy. Current Biology 2011 SEP 27 2011;21(18):1543-1547.

(30) Holmes J. Baby Brain: Training Executive Control in Infancy. Current Biology 2011 9/27;21(18):R684-R685.

(31) Wass SV. Applying cognitive training to target executive functions during early development. Child Neuropsychology 2015 MAR 4 2015;21(2):150-166.

(32) Wass SV, Scerif G, Johnson MH. Training attentional control and working memory – Is younger, better? Developmental Review 2012 12;32(4):360-387.

(33) Spittle A, Orton J, Anderson PJ, Boyd R, Doyle LW. Early developmental intervention programmes provided post hospital discharge to prevent motor and cognitive impairment in preterm infants. The Cochrane database of systematic reviews 2015 Nov 24;11:CD005495-CD005495.

(34)Cocks K, Torgerson DJ. Sample size calculations for pilot randomized trials: a confidence interval approach. Journal of clinical epidemiology 2013; 66(2): 197-201.

(35) Gartstein MA, Rothbart MK. Studying infant temperament via the revised infant behavior questionnaire. Infant Behavior and Development 2003;26(1):64-86.

(36) Wass, S. V., Cook, C., & Clackson, K. (2017). Changes in behavior and salivary cortisol after targeted cognitive training in typical 12-month-old infants. *Developmental psychology*, *53*(5), 815-825.
