# Supplementary material for: Attention and social communication skills of very preterm infants after training attention control: Bayesian analyses of a feasibility study
Source: PLoS One. 2022 Sep 22;17(9):e0273767. doi: 10.1371/journal.pone.0273767 (PMC9499320; doi:10.1371/journal.pone.0273767)
Supplement: Attachment — Submitted filename: comments.doc [file pone.0273767.s005.doc]

**Review comments on Manuscript Number: (PONE-D-21-39854) entitled '' Attention and Social Communication Skills of Very Preterm Infants after Training Attention Control: Findings from a Feasibility Study''.**

I believe this is potentially an interesting and useful research. Overall, this study provides a novel approach.  However, the following concerns will need to be addressed.

**Title:**

Well structured

**Abstract**

- It is highly recommended to write the abstract in more structured way (background, methods, results, conclusion)
- It is recommended to write keywords (5 to 7 keywords).

**Background (the Introduction)**

- Well structured.

**Methods**

- Satisfactory

**Statistical analysis**

- Satisfactory

**Discussion**

- Satisfactory
- The limitations should be clarified in a separate section at the end of discussion.

**References**

- Some references need to be updated
