# Supplementary material for: Attention and social communication skills of very preterm infants after training attention control: Bayesian analyses of a feasibility study
Source: PLoS One. 2022 Sep 22;17(9):e0273767. doi: 10.1371/journal.pone.0273767 (PMC9499320; doi:10.1371/journal.pone.0273767)
Supplement: Attachment — Submitted filename: Response to Reviewers.docx [file pone.0273767.s006.docx]

**Manuscript Number: (PONE-D-21-39854)**

**Title: '' Attention and Social Communication Skills of Very Preterm Infants after Training Attention Control: Findings from a Feasibility Study''.**

## Reviewer #1:

I believe this is potentially an interesting and useful research. Overall, this study provides a novel approach. However, the following concerns will need to be addressed.

Title:

Well structured

Abstract

- It is highly recommended to write the abstract in more structured way (background, methods, results, conclusion)

***Answer: We thank the reviewer for the positive feedback and the comments. We have revised the Abstract (Manuscript Page 2) as suggested.***

- It is recommended to write keywords (5 to 7 keywords).

***Answer: We have provided a list of keywords after the Abstract (see Manuscript, Page 3).***

Background (the Introduction)

- Well structured.

Methods

- Satisfactory

Statistical analysis

- Satisfactory

Discussion

- Satisfactory

- The limitations should be clarified in a separate section at the end of discussion.

***Answer: We have clearly identified the section on limitations providing a sub-title and moving this at the end of the Discussion, as suggested (Manuscript, Page 26).***

References

- Some references need to be updated

***Answer: We updated these. In addition, we have provided references to recent key papers such as: Kaul et al. 2021, DOI: 10.1038/s41390-021-01895-8; Wheelock et al. 2021, DOI: 10.1093/cercor/bhaa303; Eves et al., 2021, DOI: 10.1097/DBP.0000000000000806.***

## Reviewer #2

Reviewer #2: The paper submitted by the authors is interesting and well-written. The statistical analysis is well-done and the results are illustrated in a good way. Therefore, the paper can be accepted in its current form.

***Answer: We thank the reviewer for the praise received and their time in reviewing our manuscript.***

## Reviewer #3

The manuscripts looks to be good but have some feedbacks for minor correction:

1. in short title: Better not use the abbreviation VP.

***Answer: We thank the reviewer for the positive feedback. We have accepted this suggestion and corrected the short title.***

2. Usually keywords are <6.

***Answer: We have provided a list of keywords after the Abstract (Manuscript, Page 3).***

3. Bayesian analysis is more explained in introduction which need to be in method section.

***Answer: We have thought carefully about this comment. We have however come to the conclusion that a presentation of the key principle underlying Bayesian approaches and how these differ from more traditional approaches is necessary because: (a) The manuscript aims to make a case for the former approaches being used more widely, particularly in exploratory and small-sample analyses; (b) Bayesian approaches are still not widely recognised across disciplines. We thus believe that a relatively short presentation (2 paragraphs) of Bayesian approaches in the Introduction contributes to clarity and dissemination of the paper among a wider audience.***

4. Sample size is very small to generalize the study findings.

***Answer: We have further emphasised and highlighted this point in the “Limitations” section in the Discussion (Page 26)***

5. Will you clarify the intervention received by control group?

***Answer: We have restructured the “Interventions” section in the Methods (Page 9 and 10) to increase clarity and provide more details about the control procedure.***

6. It would be good to start the 1st paragraph of the discussion with aim and hypothesis .

***Answer: Thank you for this comment, which we have taken into account, revising the Discussion accordingly (Page 23).***

7. In line 302 to 307, 322 it would be compare and contras the findings with other study.

***Answer: We have provided some comparisons to two studies that have used similar procedures in the delivery of the intervention as well as similar measures, in Page 23, as suggested.***

8. Though the participants were of corrected age of <12 months, what other criteria were there for inclusion? like delay developmental milestones or normal hand function like gripping .....

***Answer: We have highlighted these criteria more clearly in the Methods section (Page 6). In the Limitations section of the Discussion (Page 26) we have also highlighted that the inclusion/exclusion criteria determined the sample was made up of healthy VP infants.***
